# Efficacy of hydrolyzed collagen injections compared to platelet-rich plasma and hyaluronic acid in the treatment of patients with symptomatic knee osteoarthritis: a retrospective clinical study

**DOI:** 10.1186/s12891-025-08811-9

**Published:** 2025-07-04

**Authors:** Angel Alberto Heredia Sulbaran, Pablo Alonso Gomez

**Affiliations:** Infanta Elena Hospital, Carretera Sevilla s/n, Huelva, 21080 Spain

**Keywords:** Knee osteoarthritis, Hydrolyzed collagen, Cartilage, Hyaluronic acid, Platelet-rich plasma, Osteoarthritis

## Abstract

**Background/Objectives:**

Hydrolyzed collagen (CHG) is an emerging infiltrative therapy for the treatment of osteoarthritis. The objective of this retrospective study was to compare its effectiveness with that of hyaluronic acid (HA) and platelet-rich plasma (PRP).

**Methods:**

The Western Ontario and McMaster Universities Osteoarthritis Index (WOMAC) scores of 72 patients with Kellgren-Lawrence grade 1–4 osteoarthritis were recorded and analyzed over a 12-month follow-up period. Patients received either three CHG injections, a single HA injection, or three PRP injections and the results were compared. Of the participants, 23 were treated with CHG, 25 with HA, and 24 with PRP.

**Results:**

Treatment effectiveness was assessed at 3, 6, and 12 months. At the 12-month follow-up, the CHG group showed a 56% improvement in the WOMAC total score, compared to 22.5% for the HA group and 47% for the PRP group. Pain reduction was also greatest in the CHG group, with a 52% decrease at 12 months, versus 16% in the HA group (*p* < 0.05).

**Conclusions:**

In this retrospective study, CHG demonstrated a more sustained therapeutic effect in terms of pain relief and functional improvement compared to HA and PRP over a one-year period. No side effects were observed in any of the treatment groups.

## Background

The world’s population is ageing, and a recent study estimated that over 1.6 billion adults have a condition that could benefit from rehabilitation, with musculoskeletal disorders accounting for the vast majority of these cases [1].

Osteoarthritis (OA) is a severely debilitating musculoskeletal disorder whose prevalence is rising sharply worldwide, resulting in significant societal and economic costs [1, 2].

It affects several joints and involves both the cartilage and surrounding tissues. Among the joints most commonly affected—along with the hip, proximal and distal interphalangeal joints, and the facet joints of the spine—the knee is one of the most frequently involved [3, 4]. Clinical manifestations of OA may result from synovial fluid accumulation, bony deformity of the joints, or active inflammation [3, 4].

Cartilage regenerative therapies are still in their early stages and have not yet become routine practice. Current treatment for less severe cases primarily focuses on relieving symptoms and improving patients’ quality of life [5, 6]. Therefore, management of cartilage damage typically includes pharmacological treatment, non-pharmaceutical interventions, and, in more advanced cases, surgical procedures [5]. Pharmaceuticals play a major role when OA becomes symptomatic and painful. A variety of treatments are currently used to reduce discomfort, including corticosteroids, hyaluronic acid (HA) in different molecular weights, and biological agents such as platelet-rich plasma (PRP) or mesenchymal stem cell injections [7, 8]. Recent meta-analyses have shown that PRP is more effective than HA at both 6- and 12-month follow-up intervals [9, 10]. However, another meta-analysis comparing PRP to placebo in patients with mild to moderate OA found no significant difference in symptom relief or joint structure [11]. These conflicting findings may stem from the wide variety of PRP preparation kits, which can result in inconsistent clinical outcomes.

Furthermore, the use of corticosteroid injections has been linked to an increased risk of knee arthroplasty in patients with symptomatic knee OA [12]. Conversely, biological treatments—especially when combined with other therapeutic approaches—have shown greater efficacy in treating OA. They effectively reduce symptoms while improving patients’ quality of life [13].

Since OA leads to progressive degradation of both the extracellular matrix and joint collagen, exogenous collagen supplementation has also been explored as an alternative or adjuvant treatment in painful cases with functional loss [14]. A recent review highlighted the positive effects of various injectable collagen formulations, suggesting their ability to promote extracellular matrix deposition by chondrocytes [14]. In recent years, a novel injectable collagen formulation—low molecular weight type I hydrolyzed bovine collagen (Chondrogrid^®^, Bioteck S.p.A., Arcugnano, Italy; CHG)—has been administered via intra-articular injections, with excellent results reported in the treatment of patients with symptomatic knee OA [15, 16].

Although promising outcomes have been reported for the OA treatment with collagen formulations, no comparative studies involving HA or PRP are currently available. Therefore, this retrospective study aims to evaluate the efficacy and safety of CHG in comparison to HA and PRP in patients with symptomatic knee OA.

## Materials and methods

This retrospective study was conducted in accordance with the Declaration of Helsinki and approved by the Ethics Committee of FABIS (Fundación Andaluza Beturia para la Investigación en Salud; protocol number AHS-COL-2021-01). Informed consent was obtained from all patients.

Clinical records of all consecutive patients who presented to the Orthopaedic Department of Infanta Elena Hospital (Huelva, Spain) between February 2021 and May 2021 for symptomatic knee osteoarthritis (OA) were retrospectively reviewed. All patients received treatment with either CHG (ChondroGrid^®^, Bioteck S.p.A., Arcugnano, Italy), HA (ADANT^®^, Meiji Pharma Spain, S.A., Spain), or PRP (Class I per the Ehrenfest classification [17]), prepared using the closed technique (Endoret^®^, BTI Biotechnology Institute, Spain).

Inclusion criteria were: a) diagnosis of knee OA graded 1 to 4 according to the Kellgren and Lawrence scale; b) treatment with CHG, HA, or PRP; c) age over 18 years.

Exclusion criteria, applied to avoid bias in the evaluation of results, included: (a) any comorbid condition that could interfere with assessment of knee symptoms or function (e.g., rheumatoid or psoriatic arthritis, fibromyalgia, Reiter’s syndrome, ankylosing spondylitis, sarcoidosis, amyloidosis); (b) active knee or skin infections; (c) prior treatment with corticosteroids or intra-articular injections (steroids, PRP, HA, or other formulations) within the previous three months; (d) surgical procedures in the previous six months; (e) presence of neoplastic disease, HIV, or HCV; f) history of drug or alcohol abuse.

CHG consists of 4 mg of low molecular weight (< 3 kDa) type I hydrolyzed bovine collagen. It is supplied as a freeze-dried powder that must be dissolved in 2 ml of water for injections before use, according to the instructions for use. Patients in the CHG group received three intra-articular injections (each 2 ml, containing 4 mg of collagen): the first at baseline, the second after 15 days, and the third approximately one month later. The HA used is a sterile 1% sodium hyaluronate solution obtained from *Streptococcus zooepidemicus* via fermentation and subsequent purification. Its molecular weight ranges from 600 to 1200 kDa. HA is provided in a ready-to-use syringe, and a single injection was administered after baseline assessment, as per the instructions for use.

The PRP system employed allows for a 2- to 3-fold increase in the patient’s baseline platelet concentration. PRP was administered in three weekly intra-articular injections over three consecutive weeks, in accordance with the product’s instructions for use.

Immediately before each injection—and at 3-, 6-, and 12-month follow-ups from the last injection—the following data were collected: demographic information (age, gender, BMI), chronic conditions, OA severity (Kellgren and Lawrence score), and Western Ontario and McMaster Universities Osteoarthritis Index (WOMAC) scores for pain, stiffness, and physical function. Adverse events were also recorded in the clinical notes.

### Statistical analysis

All statistical analyses were performed using Origin(Pro), Version 2019 (OriginLab Corporation, Northampton, MA, USA). Continuous variables with normal distribution were reported as mean ± standard deviation (SD), while categorical variables were expressed as absolute numbers and percentages. Normality of distribution was assessed using the Kolmogorov–Smirnov test. Normally distributed variables were compared using unpaired *t*-tests. Repeated measures analysis of variance (ANOVA) was used to evaluate changes in the WOMAC score and its three subscales (pain, stiffness, physical function) across time points (baseline, 3 months, 6 months, and 12 months). When appropriate, Bonferroni post-hoc correction was applied. Statistical significance was set at *p* ≤ 0.05. In Tables 1, 2 and 3, percentage improvement was calculated using the following formula:

$$\left( {\left( {Baseline\,-\,Visit} \right)\,/\,Baseline} \right)\, \times \,100$$

## Results

Clinical records of seventy-two consecutive patients who presented to the Orthopaedic Department of Infanta Elena Hospital (Huelva, Spain) between February and May 2021 for symptomatic knee osteoarthritis (OA) were retrospectively reviewed. All patients had received intra-articular treatment with one of the following injectable therapies: hydrolyzed collagen (CHG), hyaluronic acid (HA), or platelet-rich plasma (PRP).

The mean age of the study population was 47 ± 10.6 years, and 41 patients (56.9%) were male (Table 4). Most patients had no comorbidities, although 8 (11%) had hypertension and 3 (4.2%) were diabetic. Regarding treatment distribution, 23 patients (32%) received CHG, 25 (34.7%) received HA, and 24 (33.3%) were treated with PRP (Table 1).

**Table 1:** Baseline demographic and clinical characteristics of the study population

Demographic features	Value
Total number of patients	72
Age (years), mean (SD)	47 (10.6)
Gender (male), n (%)	41 (56.9)
Body Mass Index, mean (SD)	29.9 (4.1)
Hypertension, n (%)	8 (11.0)
Diabetes, n (%)	3 (4.2)
Rheumatoid arthritis, n (%)	1 (1.4)
Hypothyroidism, n (%)	1 (1.4)
Kellgren-Lawrence scale-CHG, mean (SD)	2.2 (0.8)
Kellgren-Lawrence scale-HA, mean (SD)	2.2 (1.1)
Kellgren-Lawrence scale-PRP, mean (SD)	2.6 (0.7)
Patients treated with CHG, n (%)	23 (32)
Patients treated with HA, n (%)	25 (34.7)
Patients treated with PRP, n (%)	24 (33.3)

### CHG group

**Fig. 1:**
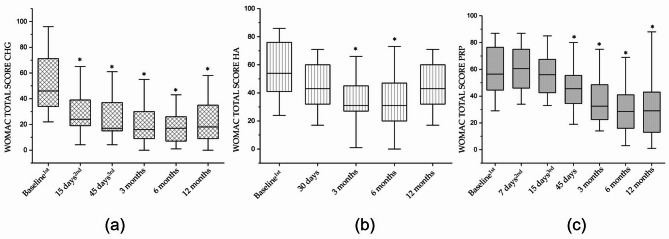
Box plot representing the WOMAC Total Score for CHG (a), HA (b), and PRP (c). Each box shows the interquartile range (IQR); the black horizontal line within the box indicates the median. The upper and lower whiskers represent the maximum and minimum values, respectively. An asterisk (*) denotes a statistically significant intra-group difference compared to baseline (*p* < 0.05). The superscripts 1st, 2nd, and 3rd indicate the number of infiltrations for each treatment at the corresponding time-point

**Fig. 2:**
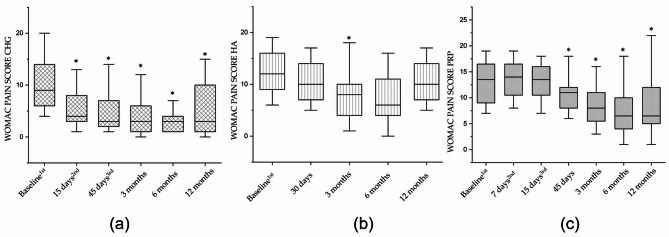
Box plot representing the WOMAC Pain Score for CHG (**a**), HA (**b**), and PRP (**c**). Each box shows the interquartile range (IQR); the black horizontal line within the box indicates the median. The upper and lower whiskers represent the maximum and minimum values, respectively. An asterisk (*) denotes a statistically significant intra-group difference compared to baseline (*p* < 0.05). The superscripts 1st, 2nd, and 3rd indicate the number of infiltrations at the corresponding time-point

**Fig. 3:**
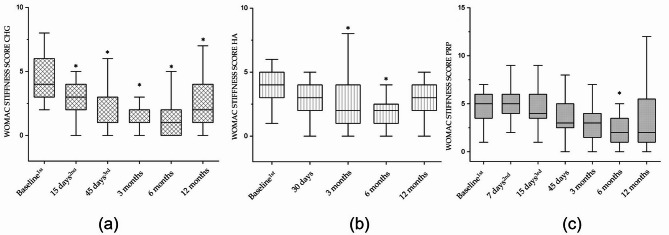
Box plot representing the WOMAC Stiffness Score collected for CHG (**a**), HA (**b**), and PRP (**c**). Each box represents the interquartile range (IQR); the median is the black horizontal line inside the IQR. The upper and lower whiskers represent the maximum and minimum values of the population, respectively. The asterisk (*) indicates a statistically significant intra-group difference compared to the baseline (*p* < 0.05). The superscripts 1st, 2nd, and 3rd indicate the number of infiltrations at the corresponding time-point

**Fig. 4:**
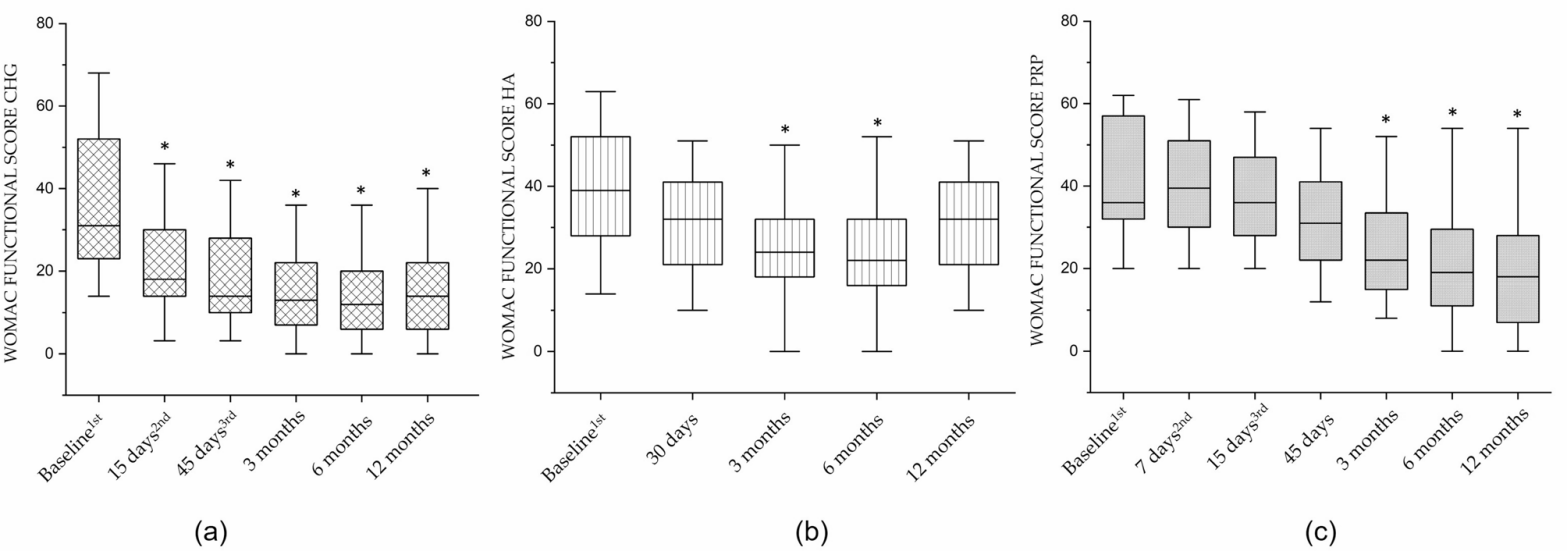
Box plot representing the WOMAC Functional Score collected for CHG (**a**), HA (**b**), and PRP (**c**). Each box represents the interquartile range (IQR); the median is the black horizontal line inside the IQR. The upper and lower whiskers represent the maximum and minimum values of the population, respectively. The asterisk (*) indicates a statistically significant intra-group difference compared to the baseline (*p* < 0.05). The superscripts 1st, 2nd, and 3rd indicate the number of infiltrations at the corresponding time-point

Twenty-three patients in the study were treated with CHG. The WOMAC total score showed that CHG was effective after the first injection, with an improvement of 44% (*p* < 0.001). Additional improvement was observed following the second and third injections (see Table 1). Subsequent follow-ups confirmed that this improvement was maintained up to 12 months (Fig. 1a; Table 1), with statistically significant differences compared to baseline (*p* < 0.001). The WOMAC subscales for pain, stiffness, and physical function showed a similar trend. In particular, pain and stiffness improved by 48% and 53% (*p* < 0.05) respectively after the first injection, with further gains after the second and third injections (Figs. 2a and 3a; Table 1). Functional improvement reached 43% (*p* < 0.001) after the first injection and increased to 58% (*p* < 0.001) at the 12-month follow-up (Fig. 4a; Table 2).

**Table 2:** Intra-group comparison of WOMAC scores for patients treated with CHG at different time points. The superscripts *1st*, *2nd*, and *3rd* indicate the number of infiltrations received at each corresponding time point. Asterisks (*) and bolded values indicate statistically significant differences (*p* < 0.05)

Follow-up	WOMAC score total, mean (SD)	% Total Improvement compared to baseline (*p*-value)	Pain score, mean (SD)	% Pain Improvement compared to baseline (*p*-value)	Stiffness score, mean (SD)	% Stiffness Improvement compared to baseline (*p*-value)	Physical function score, mean (SD)	% PhysicalImprovement compared to baseline (*p*-value)
**Baseline** ^**1st**^	51.4 (23.3)	NA	10.2 (4.6)	NA	4.4 (1.9)	NA	36.4 (15.5)	NA
**15 days** ^**2nd**^	28.7 (15.2)	44% (< 0.001)*	5.3 (3.3)	48% (0.001)*	2.1 (1.4)	53% (0.02)*	21.2 (10.7)	43% (< 0.001)*
**45 days** ^**3rd**^	24.5 (15.5)	52% (< 0.001)*	4.7 (3.7)	53% (< 0.001)*	1.7 (1.4)	63% (< 0.001)*	18.1 (10.8)	50% (< 0.001)*
**3 months**	19.7 (14.2)	61% (< 0.001)*	4 (3.7)	61% (< 0.001)*	1.4 (1.3)	68% (< 0.001)*	14.3 (10.1)	61% (< 0.001)*
**6 months**	18.4 (14)	64% (< 0.001)*	3.7 (3.7)	64% (< 0.001)*	1.4 (1.4)	69% (< 0.001)*	13.4 (10)	63%(< 0.001)*
**12 months**	22.8 (17.8)	56% (< 0.001)*	4.9 (5.1)	52% (< 0.001)*	2.5 (2.1)	44% (0.003)*	15.4 (11.3)	58%(< 0.001)*

### HA group

Twenty-five patients in the study were treated with HA. The WOMAC total score showed a statistically significant improvement of 36% at the 3-month follow-up compared to baseline (*p* < 0.01; Fig. 1b; Table 2). However, this improvement was not maintained at 12-month follow-ups, where scores were no longer significantly different from baseline. The WOMAC pain subscore improved significantly by 33% at 3 months (*p* < 0.05), but the effect diminished to 16% at 12 months (Fig. 2b; Table 2). The stiffness subscore demonstrated a 47% improvement at 6 months (*p* < 0.01), which declined to 30% at 12 months (Fig. 3b; Table 2). Likewise, the functional subscore improved significantly by 40% at 6 months (*p* < 0.001), but this benefit dropped to 24% by the 12-month follow-up (Fig. 4b; Table 3).

**Table 3:** Intra-group comparison of WOMAC scores for patients treated with HA at different time points. The superscript “1st” indicates the number of infiltrations at the corresponding time point. Asterisks (*) and bolded values indicate statistically significant differences (*P* < 0.05)

Follow-up	WOMAC score total, mean (SD)	% Total Improvement compared to baseline (*p*-value)	Pain score, mean (SD)	% Pain Improvement compared to baseline (*p*-value)	Stiffness score, mean (SD)	% Stiffness Improvement compared to baseline (*p*-value)	Physical function score, mean (SD)	% PhysicalImprovement compared to baseline (*p*-value)
**Baseline** ^**1st**^	56.5 (19.6)	NA	12.3 (4.2)	NA	4 (1.6)	NA	40.2 (14.8)	NA
**30 days**	39.2 (20.6)	31% (0.17)	9 (4.7)	27% (1)	2.4 (2)	39% (0.2)	27.8 (14.7)	31% (0.13)
**3 months**	36 (20.9)	36% (0.002)*	8.3 (5.2)	33% (0.02)*	2.5 (2.3)	37% (0.04)*	25.2 (14.6)	37% (0.002)*
**6 months**	33.6 (19.5)	40% (< 0.001)*	7.6 (4.9)	38% (0.002)*	2.3 (1.9)	47% (0.004)*	24 (13.5)	40% (< 0.001)*
**12 months**	43.8 (15.9)	22.5% (0.17)	10.3 (3.9)	16% (1)	2.8 (1.5)	30% (0.2)	30.8 (11.7)	24% (0.13)

### PRP group

Twenty-four patients in the study were treated with PRP. The WOMAC total score indicated no significant improvement up to 45 days after the start of treatment. However, a progressive improvement was observed thereafter, reaching a 47% improvement at 12 months (*p* < 0.001; Fig. 1c; Table 3). The WOMAC pain score followed a similar trend, showing a 35% improvement at the 12-month follow-up (Fig. 2c; Table 3). Regarding stiffness, PRP injections resulted in a statistically significant 48% improvement at 6 months (≤ 0.01), which decreased to 27% by 12 months (Fig. 3c; Table 3). Functional scores improved significantly by 38% at 3 months (*p* < 0.01) and continued to increase, reaching 51% at the 12-month follow-up (Fig. 4c; Table 4).

The varied effects of PRP across the WOMAC subdomains may reflect different underlying biological mechanisms. In the long term, stiffness (27%) appears to be less responsive to PRP than pain relief (35%) and physical function (51%). This variation may be linked to patient-specific factors, which require further investigation.

**Table 4:** Intra-group comparison of WOMAC scores for patients treated with PRP at different time-points. The superscripts 1st, 2nd and 3rd indicate the number of infiltrations at the corresponding time-point. Asterisks (*) and bolded values indicate statistically significant differences (*P* < 0.05)

Follow-up	WOMAC score total, mean (SD)	% Total Improvement compared to baseline (*p*-value)	Pain score, mean (SD)	% Pain Improvement compared to baseline (*p*-value)	Stiffness score, mean (SD)	% Stiffness Improvement compared to baseline (*p*-value)	Physical function score, mean (SD)	% PhysicalImprovement compared to baseline (*p*-value)
**Baseline** ^**1st**^	60.4 (19.2)	NA	13.1 (3.9)	NA	4.5 (1.7)	NA	41.6 (13.7)	NA
**7 days** ^**2nd**^	59.6 (16.4)	-0.6% (1)	13.8 (3.3)	-5.4% (1)	5.2 (1.8)	-15% (1)	50.1 (13.1)	-20% (1)
**15 days** ^**3rd**^	55.3 (15.1)	6.6% (1)	13 (3.3)	1% (1)	4.8 (2)	-7% (1)	37.5 (11.6)	10% (1)
**45 days**	45.8 (15.2)	23% (0.25)	10.5 (3.4)	20% (0.59)	3.5 (1.7)	22% (1)	31.8 (11.6)	24% (0.25)
**3 months**	37.4 (18)	37% (0.001)*	8.6 (4)	35% (0.004)*	2.9 (1.9)	36% (0.36)	26 (13)	38% (0.002)*
**6 months**	30.8 (19.9)	48% (< 0.001)*	7.5 (4.3)	43% (< 0.001)*	2.3 (1.9)	48% (0.01)*	21 (14)	49% (< 0.001)*
**12 months**	32.4 (23.5)	47% (< 0.001)*	8.5 (5.7)	35% (< 0.001)*	3.3 (3)	27% (0.83)	20.6 (15.3)	51% (< 0.001)*

### Inter-group differences among CHG, HA and PRP treatments

Improvement in the total WOMAC score was statistically different among the three treatments (Table 5). CHG resulted in a more sustained effect compared to both HA and PRP over a 12-month period. Specifically, CHG was significantly more effective than HA at every time point and showed greater efficacy than PRP up to the 3-month follow-up.

The overall reduction in pain at each follow-up after the last injection was significantly greater in patients treated with CHG (*p* < 0.05) compared to both HA and PRP. At the 12-month follow-up, pain severity was reduced by 52% with CHG, 16% with HA, and 35% with PRP (Table 5). Similarly, improvement in stiffness at 12 months was 44% for CHG, 30% for HA, and 27% for PRP (Table 5). Functional improvement at each follow-up after the last injection was also significantly higher in the CHG group compared to HA (*p* < 0.05). At 12 months, physical function improved by 58% with CHG, 24.4% with HA, and 51% with PRP (Table 5).

**Table 5:** Inter-group comparison of WOMAC scores for patients treated with CHG, HA, and PRP at different time points. Asterisks (*) indicate statistically significant differences (*p* < 0.05)

	Follow-up	CHG	HA	PRP	*P* value CHG vs. HA	*P* value CHG vs. PRP	*P* value PRP vs. HA
WOMAC score total, mean (SD)	baseline	51.4 (23.3)	56.5 (19.6)	60.4 (19.2)	1	0.39	1
	3-months	19.7 (14.2)	36 (20.9)	37.4 (18)	0.007*	0.003*	1
	6-months	18.4 (14)	33.6 (19.5)	30.8 (19.9)	0.014*	0.063	1
	12-months	22.8 (17.8)	43.8 (15.9)	32.4 (23.5)	0.001*	0.28	0.12
*% improvement at 12 months FU*		*56%*	*22.5%*	*47%*			
Pain score, mean (SD)	baseline	10.2 (4.6)	12.3 (4.2)	13.1 (3.9)	0.25	0.06	1
	3-months	4 (3.7)	8.3 (5.2)	8.6 (4)	0.003*	0.002*	1
	6-months	3.7 (3.7)	7.6 (4.9)	7.5 (4.3)	0.007*	0.011*	1
	12-months	4.9 (5.1)	10.3 (3.9)	8.5 (5.7)	< 0.001*	0.042*	0.6
*% improvement at 12 months FU*		*52%*	*16%*	*35%*			
Stiffness score, mean (SD)	baseline	4.4 (1.9)	4 (1.6)	4.5 (1.7)	1	1	0.83
	3-months	1.4 (1.3)	2.5 (2.3)	2.9 (2)	0.19	0.04*	1
	6-months	1.4 (1.4)	2.3 (1.9)	2.1 (2)	0.66	0.27	1
	12-months	2.5 (2.1)	2.8 (1.5)	3.3 (3)	1	0.84	1
*% improvement at 12 months FU*		*44%*	*30%*	*27%*			
Physical function score, mean (SD)	baseline	36.4 (15.5)	40.2 (14.8)	41.6 (13.7)	1	0.68	1
	3-months	14.3 (10.1)	25.2 (14.6)	26 (13)	0.012*	0.008*	1
	6-months	13.4 (10)	24 (13.5)	21 (14)	0.016*	0.13	1
	12-months	15.4 (11.3)	30.8 (11.7)	20.6 (15.3)	< 0.001*	0.54	0.026*
*% improvement at 12 months FU*		*58%*	*24%*	*51%*			

## Discussion

It has been a long time since the oral administration of hydrolyzed collagen was positively evaluated as a food supplement to support pain relief and functional recovery [18–24]. In studies with mice fed 14 C radio-labeled hydrolyzed collagen, collagen peptides rich in glycine and proline were found to reach articular cartilage within a few hours of administration [20]. It is important to note that collagen is the main constituent of the extracellular matrix of both cartilage and tendons [25–27]. Collagen’s triple helical structure is formed by multiple hydroxyproline-proline-glycine repeats [28]. Recently, synthetic hybridizing collagen peptides (CHP) containing these repeats were shown to deposit on damaged collagenic helical structures, promoting their repair [29, 30]. Therefore, injectable hydrolyzed collagen (CHG) may act by directly reinforcing the extracellular matrix of cartilage, tendons, and ligaments, which are primarily composed of collagen [31–33]. By targeting degraded collagen structures, CHG helps fill gaps in denatured sequences and promotes their reorganization, without exerting direct biological activity.

Injectable hydrolyzed collagen is emerging as a treatment for multiple musculoskeletal disorders, including osteoarthritis and tendinopathies [15, 16, 34]. Two observational studies have demonstrated its safety and efficacy in treating 90 knee osteoarthritis (OA) patients with an average Kellgren-Lawrence (KL) score of 1.9, showing a 64% reduction in the total WOMAC score about six months after the last injection [15, 16]. Hydrolyzed collagen treatment has also proven effective in pain reduction and functional recovery in patients with rotator cuff tendinopathy [34]. In this context, the treatment effect increased progressively, with pain reduction exceeding 70% during movement and more than 90% at rest and during the night at six months follow-up. Additionally, Constant and Simple Shoulder Test scores improved by 32% and 61%, respectively [34].

In this clinical study, the efficacy and safety of three hydrolyzed collagen injections were compared for the first time to the use of PRP and a single-injection hyaluronic acid (HA) treatment over a 1-year follow-up period. HA demonstrates a longer degradation time at the injection site compared to PRP and hydrolyzed collagen (CHG), where degradation occurs more rapidly. As a result, the HA Instructions for Use recommend a single injection, as its prolonged persistence at the treatment site aligns with the higher number of injections required for the other two products.

In the CHG group, there was a significant improvement in all WOMAC scores: total score, pain, stiffness, and functional recovery (see Figs. 1a, 2a, 3a and 4a, and Tables 1 and 5). These improvements began with the first injection, with a 48% pain relief compared to baseline. In the HA group, significant improvements in the WOMAC total score, pain, stiffness, and functional recovery were observed at both the 3- and 6-month follow-ups (see Figs. 1b, 2b, 3b and 4b, and Tables 2 and 5). In the PRP group, significant improvements in the WOMAC total score, pain, and functional recovery were observed from the 3-month to the 12-month follow-ups. Interestingly, PRP seemed less effective at reducing knee stiffness compared to CHG and HA. It showed a significant improvement in stiffness only at the 6-month follow-up, but this effect sharply decreased by the 12-month follow-up (see Fig. 3c; Tables 2 and 5). Overall, CHG was more effective than both HA and PRP in reducing pain and improving functionality in symptomatic patients with knee OA. Specifically, the total WOMAC score for CHG was statistically more effective than HA at every time point. Furthermore, CHG was statistically more effective than PRP up to 3 months of follow-up for the total WOMAC score. In terms of pain reduction, after 1 year from the last injection, patients treated with CHG experienced a significantly greater reduction (52%) compared to HA (16%) and PRP (35%) (see Table 5). The improvements observed with CHG up to the 6-month follow-up in this study are consistent with previous studies on the intra-articular use of hydrolyzed collagen in patients with knee OA, where 6-month follow-up was the last time point [15, 16]. In this study, patients with an average KL of 2.2 showed a 64% reduction in the total WOMAC score at the 6-month follow-up, similar to the 63% reduction observed in the study by Volpi et al. [15]. The present study extended the monitoring of CHG treatment, showing that at the 12-month follow-up, a 56% improvement in the total WOMAC score compared to baseline was still evident. PRP treatment proved more effective than HA in terms of pain relief, functional recovery, and the duration of the treatment (see Figs. 1, 2, 3 and 4; Table 5). However, the disadvantages of PRP include a slower initial response from patients and higher variability in the final results compared to CHG and HA. Interestingly, a recent meta-analysis involving 1,403 knees showed that PRP was more effective than HA, but not in the first month of follow-up [9]. This finding is supported by the results presented here, where PRP did not show improvements in any measured outcome during the first month. The longer duration of pain relief and functional recovery for PRP compared to HA, as reported here, is consistent with recent meta-analyses indicating that PRP was superior to HA at both the 6-month and 12-month follow-ups [9, 10]. This is the first clinical study comparing CHG, HA, and PRP in the treatment of symptomatic patients with knee OA. Previous studies on HA in this setting have shown no significant differences when comparing intra-articular HA to corticosteroids. Skwara et al., in a randomized clinical trial comparing a single intra-articular injection of hyaluronan with triamcinolone, found no significant difference between the two treatments, with both improving pain and functional parameters [35]. In 2014, Leighton et al. conducted a large randomized clinical trial with 442 patients with knee OA and found that NASHA hyaluronic acid gel, when administered as a single intra-articular injection, was not inferior to methylprednisolone acetate in reducing the WOMAC pain score at the 12-week follow-up [36]. Subsequently, in a randomized clinical trial involving 140 patients with OA, Askari et al. found that pain and stiffness did not improve in either the HA or corticosteroid groups at any time point after the intervention. However, the Knee Injury and Osteoarthritis Outcome Score (KOOS) suggested that symptoms and daily activities improved after 3 months in both groups [7]. In addition, in a multicenter, double-blind randomized clinical trial comparing the efficacy and safety of two hyaluronan preparations with placebo, Karlsson et al. found that patients with knee OA treated with either hyaluronan preparation or placebo showed clinical improvement during the first 26 weeks. However, when data from the two hyaluronan treatments were pooled, hyaluronan showed a significantly longer duration of clinical benefit compared to placebo [37]. Given the non-inferiority of HA to cortisone, along with the potential serious systemic and non-systemic side effects of cortisone, all authors agree on recommending hyaluronate therapy, especially for prolonged treatments [7, 35–37]. While HA is commonly used in the USA, it is not supported by the National Institute for Health and Care Excellence (NICE) in the UK [8]. Regarding PRP, literature consistently supports its superiority over hyaluronate and placebo [8, 38, 39]. Three major double-blind randomized control trials have demonstrated significant benefits across all three domains of the WOMAC score with PRP [8, 38, 39]. Patel et al., in 2013, randomized 78 patients with early knee OA to receive either PRP or saline, showing that PRP effectively reduced symptoms, although clinical benefits declined after six months [39]. Smith (2016) confirmed promising results in patients with more severe knee OA, demonstrating a 78% improvement in the WOMAC score at 12 months for PRP patients, compared to 7% for controls [38]. Cole et al. (2016), in a larger study of 99 patients randomized to PRP or HA, found no statistically significant difference between the two treatments, although PRP showed slight advantages, including a reduction in intra-articular pro-inflammatory cytokines [8]. Despite promising results, PRP’s use is limited by the lack of consensus on standardization and its high cost [40, 41]. While this study presents valuable and innovative findings compared to current literature, it has some limitations. The retrospective nature of the study introduces a potential risk of bias, and the small sample size limits the generalizability of the results. Further research should involve larger patient groups and address additional variables to strengthen the conclusions. In addition, considering the distinct mechanisms of action of PRP and hydrolyzed collagen (CHG), combining the regenerative effects of PRP with the extracellular matrix reinforcement provided by hydrolyzed collagen could offer potential therapeutic benefits. The lyophilized form of CHG could potentially be dissolved directly into PRP for combined use. Another aspect for future investigation is comparing the efficacy of hydrolyzed collagen with low-molecular weight versus high-molecular weight HA, as different molecular weights may impact treatment outcomes.

## Conclusions

Our results indicate that intra-articular injections of CHG are both safe and effective for treating symptomatic knee OA. Additionally, CHG demonstrates a more sustained therapeutic effect over a 12-month period compared to other treatments, underscoring its potential as a long-term option for symptom management. Larger, randomized clinical trials are needed to further compare the effectiveness of CHG with other adjuvant treatments.

## Data Availability

Data are available upon request due to privacy and ethical considerations.

## Abbreviations

WOMAC: Western Ontario and McMaster University Osteoarthritis Index
CHG: Hydrolyzed collagen of bovine origin
HA: Hyaluronic Acid
PRP: Platelet-Rich Plasma
OA: Osteoarthritis
